# Genetic Pathways Underlying Individual Differences in Regular Physical Activity

**DOI:** 10.1249/JES.0000000000000305

**Published:** 2022-09-01

**Authors:** Eco J.C. de Geus

**Affiliations:** Department of Biological Psychology, VU University, and Amsterdam Public Health research institute, VU University Medical Center, Amsterdam, The Netherlands

**Keywords:** twin studies, family studies, heritability, genome-wide association, exercise behavior

## Abstract

Twin and family studies show a strong contribution of genetic factors to physical activity (PA) assessed by either self-report or accelerometers. PA heritability is around 43% across the lifespan. Genome-wide association studies have implied biological pathways related to exercise ability and enjoyment. A polygenic score based on genetic variants influencing PA could help improve the success of intervention programs.

**Key Points**

- Family and twin studies show that for different types of physical activity, and across device-based or self-report assessment, meta-analyses showed broad sense heritability to be around 26% for females and 35% for males in childhood, to increase to around 42% in both sexes during adolescence, and to remain around 45% throughout adulthood.
- Genetic correlations derived from multivariate twin studies and genome-wide association studies (GWAS) results suggest that the biology of exercise (train) ability and exercise enjoyment are partly underlying the heritability of physical activity.
- GWAS on physical activity have identified several replicated genetic variants and allow the computation of polygenic scores (PGS) for physical activity.
- These PGS can be used to study the causal effects of physical activity on health, to test the interaction between genetics and physical activity interventions, and to tailor physical activity interventions to individual genotypes.

## INTRODUCTION

Despite the long-standing recognition that physical inactivity is a major burden on our health care systems (1,2), the adoption of national physical activity (PA) guidelines, and governmental policies building on these guidelines by many countries (3,4), a large proportion of the population still does not engage in enough physical activity for optimal health benefits (5,6). There is even a striking stability of the percentage of sedentary individuals from 2000 to 2015 in both the adult (7) and adolescent (8,9) population. These alarmingly high levels of physical inactivity are uniformly repeated across all countries and all continents (7,8).

Why is PA so hard to change despite our many intervention efforts? Strong forces seem to intercede between the intention to be more physically active and the actual enactment, a discordance alluded to as the intention-behavior gap (10). These forces can be external to a person, including socioeconomic factors (11) and physical factors like the built environment (12), but many biological and psychological person-specific characteristics like body composition (13), exercise ability (14), enjoyment (15), and personality traits (16,17) also are at play. Whereas there is no dispute among researchers about the multifactorial determination of PA, prioritizing one or more of the many possible determinants for research (funding) does seem to divide the research community. With the acknowledgment that it is an oversimplification, two general perspectives can be found. The first “epidemiological” perspective tries to identify all determinants that explain the variance in PA encountered in a target population, even if they are not (readily) modifiable like sex, age, socioeconomic status, and genetics. The second “interventionist” perspective focuses explicitly on those determinants of variance in PA that might be successfully modified in the target population. The interventionist's perspective would criticize the epidemiological perspective for not yielding actionable scientific results and, therefore, a failure to generate impact. The epidemiological perspective would in turn point out that a focus on actionable determinants might ignore the largest sources of variation between individuals and, therefore, overpromise on the achievable increases in PA.

In this review, I aim to close the gap between these two perspectives on the theme where the divide in the focus of research on the determinants of PA seems to be the strongest: the contribution of genetic variation to differences between individuals in their regular PA habits.

**Figure 1:**
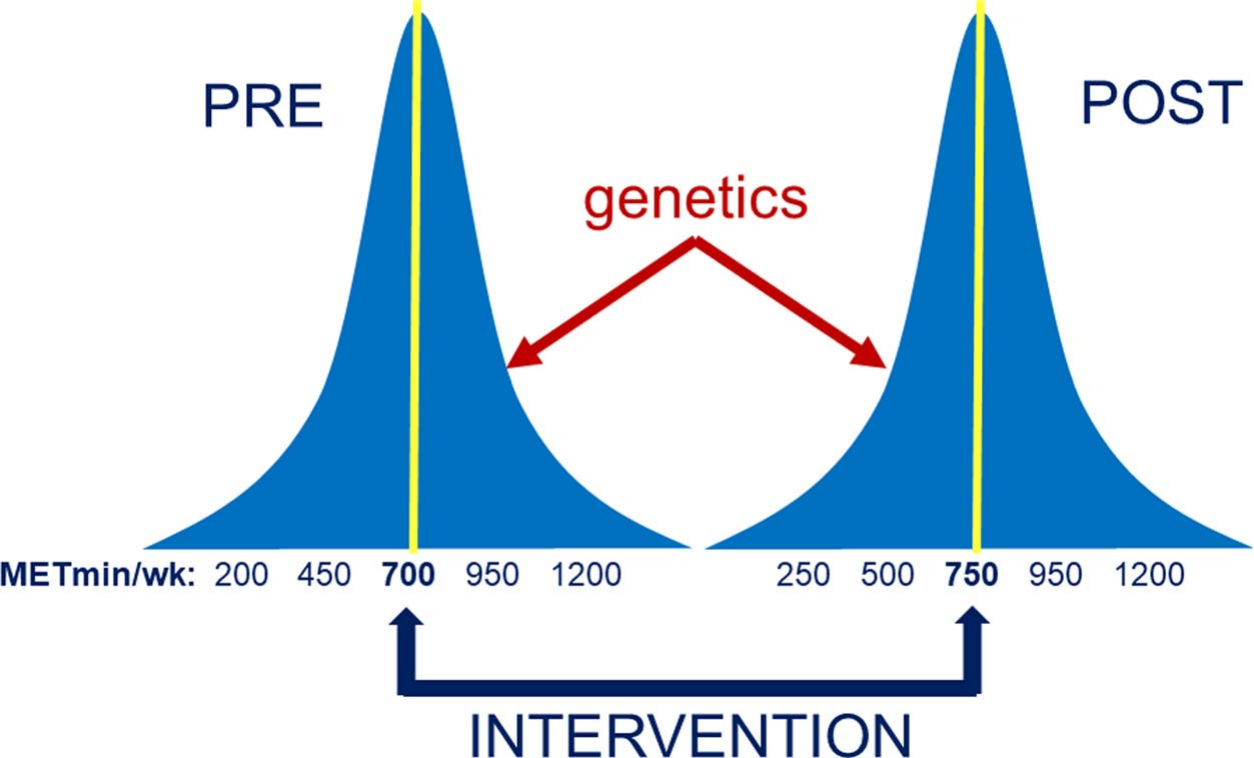
Genetics is about the variance; intervention is about the mean.

Figure 1 shows an imaginary example with the distribution of the number of METminutes spent weekly on leisure time sports and exercise activities^1^ before and after an intervention program set up to encourage more regular participation in such activities, for example, using one of the successful digital programs to increase gym visits (18). Figure 1 is aptly summarized as “intervention is about the mean, genetics is about the variance.” This means that there will be a large difference between individuals in METminutes weekly spent on exercise before, as well as after, the intervention. This is true even if the intervention is successful in raising the overall mean METminutes weekly spent on exercise. The intervention on the mean may decrease the total variance but may also increase it, because the impact of the intervention may not be uniform across individuals. If those with a high innate drive to exercise are the ones to increase their exercise levels in response to the intervention the most, the genetic variance will increase. If in contrast those with a low innate drive to exercise are most activated in response to the intervention, the genetic variance will decrease.

**Figure 2:**
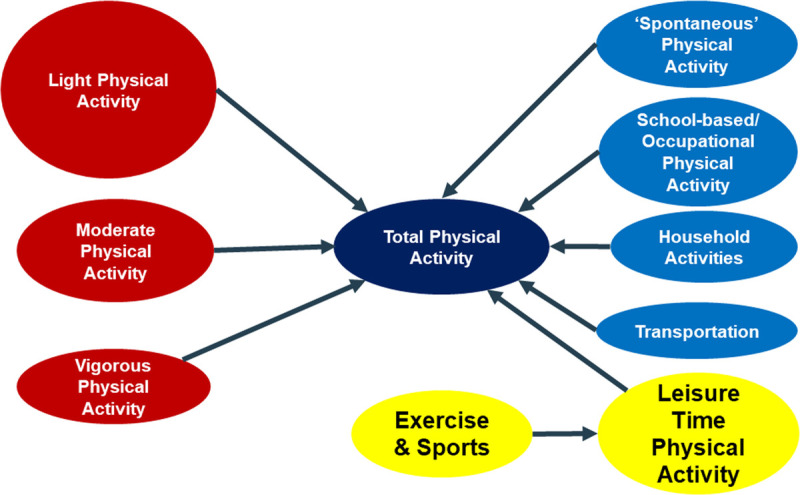
Classification of physical activity (PA) phenotypes. Note: the left hand side (red text balloons) divides total PA into different levels of energy expenditure/intensity, which are best detected by device-based or other objective measurement strategies. The right hand side (blue and yellow text balloons) divides total PA based on the context in which it occurs, which is currently still best captured by using self-report.

Figure 1 illustrates the idea that if the variance in regular sports and exercise behaviors is explained to a substantial degree by heritable factors (epidemiological perspective), this does not detract from the possibility that such behaviors can be increased by well-designed interventions (interventionist perspective). Heritability of a trait, in short, is not predestination and does not impede the development of successful intervention programs. This does not just apply to regular sports and exercise activities, which was used as the example in Figure 1, but to the full breath of PA behaviors depicted in Figure 2.

In genetic epidemiology, just as in any other area of science, findings depend strongly on the exact definition of PA or, in genetics parlance, the PA “phenotype.” A detailed discussion on the methods used to quantify PA used in the exercise genetics field is provided in Supplemental Digital Content 1, http://links.lww.com/ESSR/A60. Briefly, total physical activity (TPA) can be classified into light (LPA; 1.1 to 2.9 MET), moderate (MPA; 3.0 to 5.9 MET), and vigorous (VPA; ≥6.0 MET) activity based on fixed energy expenditure cutoffs, with the MPA and VPA often grouped together as a single moderate to vigorous physical activity (MVPA) category. When using “context” as the classifying principle, PA can be subdivided in spontaneous activity (*e.g.*, fidgeting, rocking, restless legs, pacing, shivering, tics, postural sway), occupational activity (*e.g.*, manual labor, standing at a desk, walking and lifting at work), transportation (*e.g.*, walking, cycling, skateboarding), and all leisure time physical activity (LTPA). LTPA prominently incorporates voluntary sports and exercise activities, but it also contains play for children and, for adults, hobbies like gardening, do-it-yourself home repair, or dancing. Typically, LTPA assessment is focused on moderate to vigorous activities, with a large chunk related to voluntary sports and exercise activities. Again, demonstrating substantial heritability for total daily PA or any of its subclasses shown in Figure 2 does not impede efforts to change these behaviors in a positive way. However, it would suggest large differences in the sensitivity of individuals to the current interventional strategies to which the population is already exposed. A better understanding of the pathways that lead from genetic variation to variation in PA phenotypes may help identify vulnerable subpopulations at an early age and fuel the design of tailored interventions that more effectively increase total PA or PA in specific subclasses.

In later sections, I first review and meta-analyze the current evidence for a role of genetics in the individual differences in PA phenotypes from studies comparing the PA of nontwin siblings, of parents and offspring, and of dizygotic (DZ) versus monozygotic (MZ) twins. Subclasses of PA phenotypes used are TPA, MVPA, LTPA, and voluntary exercise behavior (VEB), because these are dominant in the extant literature. Next, I review the genetic variants identified by whole-genome approaches and the biological pathways implicated by functional annotation of these variants. Finally, I address how genetics can assist us in addressing two key issues: 1) testing hypotheses on biopsychological determinants of individual differences in PA phenotypes, and 2) individual tailoring of intervention programs, for example, by using polygenetic scores for PA phenotypes.

## GENETIC CONTRIBUTION TO INDIVIDUAL DIFFERENCES IN PHYSICAL ACTIVITY

### Family Studies

Engagement in regular PA “runs in the family,” meaning that the chance of one family member being physically active increases the chance of all other family members to be, or to become, physically active. Familial resemblance of PA can be investigated by computing correlations among relatives such as siblings or parents and their offspring (Table 1). Significant correlation between related family members can be due to shared additive and nonadditive genetic factors and all environmental factors that they have in common. Additive genetic factors (A) represent the sum of all linear effects of the genetic loci that influence the trait of interest. The ratio of the variance in a trait explained by additive effects relative to the total variance is known as the narrow sense heritability. Nonadditive genetic factors (D) include intra-allelic dominance and cross-allelic interaction (epistasis) effects. The part of the total trait variance explained by the sum of additive and nonadditive genetic factors is the broad sense heritability. The common environment (C) consists of factors shared by parents and offspring (Cf), like family functioning, diet, socioeconomic status, and the neighborhood characteristics, or intragenerational factors shared by siblings (Cs), like parenting behaviors, shared peers, school, and all generation-specific factors. Twins share additional environment (Ct) including maternal behavior during pregnancy and intrauterine conditions and may also be more often in the same class or team.

**TABLE 1:** Estimating sources of familial resemblance from genetically informative designs.

Correlation Between	Caused by Familial Effects, That	Notation
Parent-offspring	Combine the sharing of the family environment (Cf) with 50% sharing of additive genetic variance	0.5*A + Cf
Siblings	Combine the sharing of the family (Cf) and sibling environment (Cs) with 50% sharing of additive genetic and up to^a^ 25% of nonadditive variance	0.5*A + ~0.25D + Cf + Cs
DZ twins	Combine the sharing of the family (Cf), shared sibling (Cs), and shared twin environments (Ct) with 50% sharing of additive genetic and up to^a^ 25% of nonadditive variance	0.5*A + ~0.25D + Cf + Cs + Ct
MZ twins	Combine the sharing of the family (Cf), shared sibling (Cs), and shared twin environments (Ct) with 100% sharing of additive genetic and 100% of nonadditive variance	A + D + Cf + Cs + Ct
Grandchild-grandparent	Consist almost exclusively^b^ of 25% sharing of additive genetic variance	0.25*A
First cousins	Consist almost exclusively^b^ of 25% sharing of additive genetic variance	0.25*A

^a^ Most sources set this to 0.25 assuming that dominance is the main source of nonadditivity. However, as explained by Keller and colleagues (19) a larger role of gene-gene interaction (epistasis) will tend to reduce this number.

^b^ Exceptions are children who spent a lot of time with their grandparents/cousins or live in the same neighborhood, which would add shared environment as a source of covariance.

**Figure 3:**
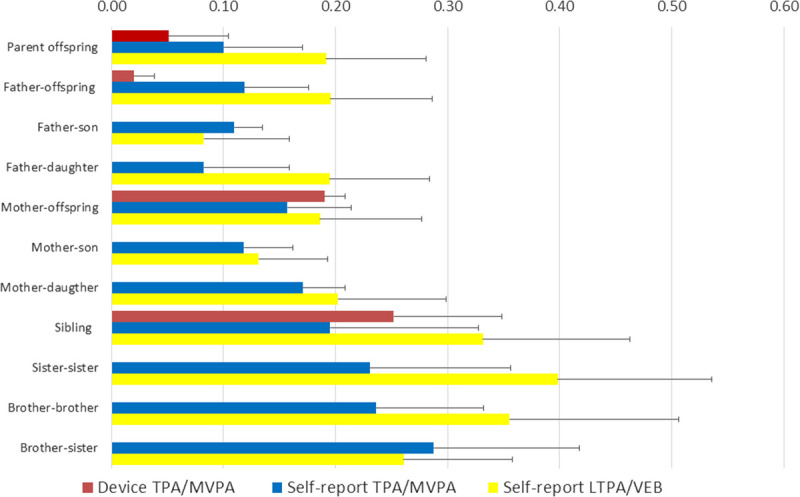
Parent offspring and sibling correlations for physical activity (PA) phenotypes. Note: Sample size–weighted mean correlations for device-based total physical activity (TPA)/moderate to vigorous physical activity (MVPA), self-reported TPA/MVPA, and leisure time physical activity (LTPA)/voluntary exercise behavior (VEB) were computed for 5098, 29147, and 137695 parent-offspring pairs, respectively, (father-offspring: 900, 10716, 65989; father-son: –, 5276, 30522; father-daughter: –, 5300, 31150; mother-offspring: 1060, 11014, 71746; mother-son: –, 5334, 32590; siblings: 4342, 10480, 33605; sister-sister: –, 1355, 10480; brother-brother: 1355, 6526; brother-sister: –, 2711, 14086). Error bars indicate the weighted SD around the correlations, derived as ∑i=1Nwixi−x¯2M−1M∑i=1Nwi.

There is now a substantive literature addressing the family-based intra- and intergenerational resemblance in TPA, MVPA, LTPA, and VEB in nuclear families (parents with multiple offspring) or larger multigeneration pedigrees. This gist of this literature is captured by Figure 3, which depicts sample size–weighted mean parent-offspring and sibling correlations from studies that reported both correlations (see Supplemental Digital Content 1, http://links.lww.com/ESSR/A60, for detailed information and references of the family studies). Figure 3 presents the correlations by assessment strategy (device-based vs self-report) and by type of PA, grouping overall (TPA/MVPA) and leisure time–based activities (LTPA/VEB).

#### Parent-offspring correlations

Parent-offspring correlations on between 5098 and 137,695 pairs range from *r* = 0.05 for device-based TPA or MVPA to *r* = 0.19 for self-reported LTPA/VEB. For TPA/MVPA, higher mother-offspring than father-offspring correlations are found, most notably for device-based MVPA/TPA in two samples with a relatively young mean offspring age. The pattern of parent-offspring correlations from the (extended) family studies in Figure 3 is congruent with the conclusions of systematic meta-analyses (20,21) that used the parent-offspring correlation as evidence for observational learning of the children through “parental modeling.” Parental modeling means that children copy the PA activity behavior of their parents, yielding a parent-offspring correlation. It is somewhat alarming that the additional possibility of genetic transmission contributing to parent-offspring correlations is not well recognized in this field of study.

#### Sibling correlations

Average full sibling correlations across the 4342 to 33,605 pairs in the studies depicted in Figure 3 were *r* = 0.19 for self-reported TPA/MVPA, *r* = 0.25 for device-based TPA/MVPA, and *r* = 0.33 for LTPA/VEB. Direct comparison of sibling correlations to the parent-offspring correlations shows that intragenerational family resemblance is about a factor two larger than intergenerational family resemblance, even though the amount of additive genetic sharing between parents and offspring and sibling pairs is the same (on average 50%). This suggests that the environment shared by the siblings but not the parents has a clear contribution to their PA resemblance, although age-specific expression of genetic factors and nonadditive genetic effects also can contribute to the lower intergenerational resemblances.

#### Heritability estimates from family studies

To estimate the specific contribution of genetic effects to familial resemblance, nuclear and extended family studies need to make specific assumptions about the shared environmental effects on PA, or they have to add information that allows the separation of genetic and shared environmental effects. Quite often shared environmental influences were simply assumed to be negligible (22,23). In other studies, familial relation were added that can be assumed to have only a limited shared environment, like grandparents and grandchildren, first cousins, and sib avuncular relations (24–27). Finally, a number of family studies enriched the parent offspring design by adding MZ and DZ twins (28,29). As can be seen in Table 1, adding either MZ twins or second- and higher-degree relations yields a series of solvable equations (“the model is identified”) for all components. Of note, the genetic component are decomposed rarely into separate additive and nonadditive effects, meaning that heritability estimates from extended family studies mostly represent “broad heritability” estimates.

Using the estimates from the family studies described in Supplemental Digital Content 1, http://links.lww.com/ESSR/A60, I computed a random effect estimate for heritability across all studies using a variance-weighted meta-analysis (30). When multiple models with different covariates were reported, I preferably selected those that only corrected for age and sex. This avoids potential collider bias arising when heritable covariates like body mass index are included in the twin or family modeling of genetic effects (31). The average heritability estimate across device-based MVPA and TPA (48%; confidence interval [CI], 30%–66%) was higher than for self-reported MVPA/TPA phenotypes (21%; CI, 14%–28%). Self-reported LTPA/VEB showed an average heritability of 29% (CI, 22%–36%). Only three studies also detected significant estimates of contribution of the common environment to PA phenotypes (24,26,27) ranging from 4% to 25%.

### Twin Studies

A more powerful design to disentangle the relative importance of shared environmental and genetic influences on a trait or behavior is the classical twin design. This design compares the intrapair resemblance between two types of twins: genetically identical twins or MZ, a result of division of a single fertilized egg during an early stage in embryonic development, and nonidentical twins or DZ, resulting from two separate fertilized eggs. When twins are reared together, the amount of sharing of a common childhood environment (Cf + Cs + Ct) is comparable for MZ and DZ twins. The important difference between MZ and DZ twins is that the former share identical genotypes, whereas the latter share on average only half of the genotypes segregating in that family. Consequently, MZ twins share 100% and DZ twins on average 50% of their additive genetic variance, and MZ twins share 100% and DZ twins on average ~25% of their nonadditive genetic variance.

If the resemblance in a PA phenotype within MZ pairs is larger than that in DZ pairs, which can be tested by comparing the MZ (rMZ) and DZ (rDZ) twin correlations, this suggests that additive genetic factors influence PA. If the MZ resemblance is more than double as large, it suggests the additional influence of nonadditive genetic factors on PA. If, however, the resemblance in the PA phenotype in DZ twins is more than half as large as it is in MZ twins, this points to the common environment as an additional cause of twin resemblance. Furthermore, the extent to which MZ twins do not resemble each other is a direct estimator of the contribution of unique environmental factors (E). These include all person-specific experiences like differential jobs or lifestyles, accidents, or other life events, and in childhood, differential treatment by the parents, going to different schools, and having nonshared friends and peers, but also somatic mutations and the stochastic part of epigenetic changes. Measurement error will also be subsumed by the unique environmental factor.

A simple set of rules of thumb can be used to estimate the contributions to the total variance in PA of the A, C, D, and E variance components (Table 2).

**TABLE 2:** Rules of thumb to estimate sources of familial resemblance from the pattern of twin correlations.

If rDZ ≤ rMZ ≤ 2*rDZ		(*e.g.*, rMZ = 0.48, rDZ = 0.30)	If rMZ > > 2*rDZ		(*e.g.*, rMZ = 0.70, rDZ = 0.25)
A	2*(rMZ − rDZ0)	.36 = 36%	A	4*rDZ − rMZ	.30 = 30%
C	2*rDZ – rMZ	.12 = 12%	C	0	.00 = 0%
D	0	.00 = 0%	D	2*rMZ – 4*rDZ	.40 = 40%
E	1 – (A + C + D)	.52 = 52%	E	1 – (A+ C + D)	.30 = 30%

Because just two covariances and the overall variance are available in a classical twin study, only three of the four A, C, D, and E factors can be tested simultaneously. The rules of thumb are therefore divided into two different scenarios. One where the MZ correlation is not larger than twice the DZ correlation, suggesting that nonadditivity (D) can be ignored, and one where the MZ correlation is substantially higher than twice the DZ correlation, suggesting that genetic nonadditivity is in play.

Applying the rules of thumb in Table 2 gives a rapid impression of (non)additive genetic and shared environmental effects, but often structural equation modeling of the full variance-covariance matrix for the PA phenotypes of DZ and MZ twins is used to estimate the contribution of the A, C/D, and E components to the total variance. Formal tests of the model fit are often used to test assumptions about equality of the means and variances in MZ and DZ, about the existence of sex differences, and to establish whether parsimonious models using just additive and unique environmental factors sufficiently explain the patterns of twin covariance.

**Figure 4:**
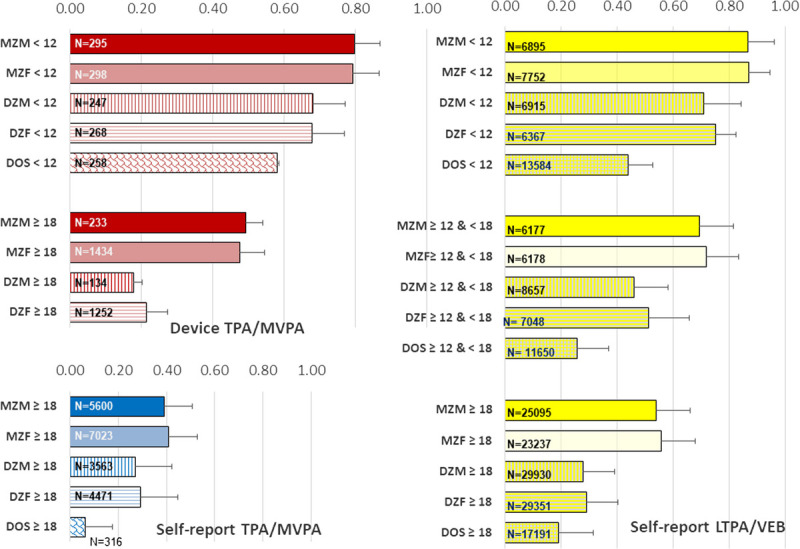
Twin correlations for physical activity (PA) phenotypes by zygosity and age group. Note: Sample-size weighted mean correlations for device-based total physical activity (TPA)/moderate to vigorous physical activity (MVPA), self-reported TPA/MVPA, and leisure time physical activity (LTPA)/voluntary exercise behavior (VEB) were computed for MZ males (MZM), DZ males (DZM), MZ female (MZF), DZ female (DZF), and DOS twin pairs in the age range of 2 to 12 years, 12 to 18 years, and older than 18. Studies were included only if they had *N* > 10 pairs in each zygosity group. Error bars indicate the weighted SD around the correlations, derived as ∑i=1Nwixi−x¯2M−1M∑i=1Nwi.

Figure 4 plots the mean sex-specific and sample size–weighted MZ and DZ twin correlations across twin studies where the mean age of the twins was less than 12 yr (childhood), between 12 and 18 yr (adolescence), and more than 18 yr (adulthood) (see Supplemental Digital Content 1, http://links.lww.com/ESSR/A60, for detailed information on the twin studies and references). Within each age group, plots are again ordered by assessment strategy and by type of PA, grouping overall (TPA/MVPA) and leisure time–based activities (LTPA/VEB).

In childhood, MZ and DZ correlations are both high, and the MZ is substantially less than twice as large as the DZ correlation, particularly for LTPA/VEB. In adolescence and adulthood, a pattern of decreasing twin correlations can be seen, but the decrease is much stronger for the DZ than the MZ twins. By far the largest amount of data is obtained by self-report, but across decent sample sizes, data also are available for device-based TPA/MVPA. Applying the rules of thumb as in Table 2, the pattern of twin correlations points to a high contribution of the shared environment that wanes from childhood to adolescence to give rise to an increasing estimated additive genetic variance as the main source of PA differences in adolescence and adulthood.

Differences between male and female same-sex twin correlations indicate quantitative sex differences, but these are relatively modest at all ages except for higher device-based female DZ correlations. Throughout the lifespan, resemblance in PA of DZ opposite sex (DOS) pairs is much lower than that in DZ same sex pairs, showing that either different genetic factors influence the PA of boys and girls or that they are exposed to sex-specific environmental influences. These cannot be modeled at the same time, but most studies, at least on VEB, have favored models with the low DOS correlations explained by different shared environmental factors for girls and boys in childhood (32,33), but by different genetic factors in adulthood (28,34).

#### Heritability estimates from twin studies

An inverse variance–weighted meta-analysis on the estimates for A and C was performed on the twin studies in Supplemental Digital Content 1, http://links.lww.com/ESSR/A60, maintaining the three age categories as before. For device-based TPA/MVPA, no sex differences in broad sense heritability estimates were found. In children younger than 12, the mean estimate for the contribution of genetic variance to total variance in device-based TPA/MVPA was 19% (95% CI, 10%–28%). Too few studies estimated the heritability of device-based TPA/MVPA in adolescents or self-reported TPA/MVPA in children and adolescents, but in twins older than 18, the heritability estimate for device-based TPA/MVPA was 54% (95% CI, 48%–59%), and for self-reported TPA/MVPA, it was 37% (95% CI, 30%–44%). As in the family studies, therefore, heritability across device-based MVPA and TPA was clearly higher than for self-reported MVPA/TPA phenotypes.

In keeping with previous reports, small but significant quantitative sex differences in heritability estimates for self-reported LTPA/VEB were found, most prominently at younger ages (35–39), and results are reported separately for males and females. For males younger than 12, the mean heritability estimate for LTPA/VEB was 36% (95% CI, 28%–43%). For females younger than 12, lower heritability estimate for LTPA/VEB are found of 24% (95% CI, 18%–30%). For males between 12 and 18, the mean heritability estimate for LTPA/VEB was 47% (95% CI, 39%–55%). For females between 12 and 18, again a lower heritability estimate of 42% (95% CI, 35%–50%) for LTPA/VEB was found, although male and female CIs largely overlapped. Above age 18, quantitative sex differences disappeared with adult heritability for joint male and female twins estimated at 48% (95% CI, 44%–52%).

#### Estimates of the role of common environment from twin studies

In parallel to the changing influence of genetic factors, we find a symmetrical change in the role of the shared environment in childhood and adolescence. For both males and females, the mean estimate for the common environmental variance in device-based TPA/MVPA was 55% (95% CI, 49%–61%) in children younger than 12, but reduced to a nonsignificant 2% in adulthood. Likewise, the common environmental variance in self-reported TPA/MVPA (3%) was not significant in adulthood.

Estimates of common environmental variance in self-reported LTPA/VEB were 51% (95% CI, 42%–60%) in male children and 62% (95% CI, 57%–67%) in female children. Common environmental influences strongly waned during adolescence, averaging 23% (95% CI, 13%–33%) in male adolescents and 28% (95% CI, 19%–38%) in female adolescents. In adulthood, little evidence for remaining effects of having shared an early environment remains for LTPA and VEB. Indeed, results from the largest study on VEB using an extended twin pedigree design (25) suggested that a shared environment by siblings (Cf + Cs, typically up until age ~18) explains 4% of the variance in adult exercise behavior, and sharing an environment by twins (Cf + Cs + Ct) explains 8%. Sharing a household by spouses yielded much higher (20%–24%) contributions to PA variance, but this effect incorporates the increasing resemblance in partners that occurs over time through marital interaction, which should be considered part of the unique environment.

A few notable exceptions to the overall trends of no or low C in adulthood deserve mention (34,40–43). These studies have in common that they used a binary PA phenotype defined as yes/no adherence to (a single type of) regular exercise or adherence to a preset criterion. The largest contribution of a common environment in adults was reported in 9654 Chinese twin pairs (43). High and almost identical MZ (*r* = 0.87) and DZ (*r* = 0.85) twin correlations were reported for PA defined as 150 min of MVPA per week. This study was so outlying that it was not used in the meta-analyses above. Further studies are needed to see if this intriguing deviant finding reflects the specific single-question phenotyping used, the relatively poor performance of the zygosity-determining questions compared with DNA testing, or a much stronger structuring environment for family members in China — most other studies being from Europe and the United States.

### Synthesis From Twin and Family Studies

The studies reviewed by meta-analyses span a total of 70,200 members in family studies and 83,694 complete twin pairs that contributed data at one or more ages and for one or more PA phenotypes. Results unanimously support a strong genetic contribution to PA, and this holds independent of design (family or twin), PA phenotype examined (TPA, LTPA, MVPA, or VEB), or method used (survey, interview, or accelerometer). This conclusion is fully congruent with earlier narrative and systematic reviews (44–48) that supported “genetics” as the monolithic determinant claiming the largest chunk of the observed interindividual variation in PA behaviors.

**Figure 5:**
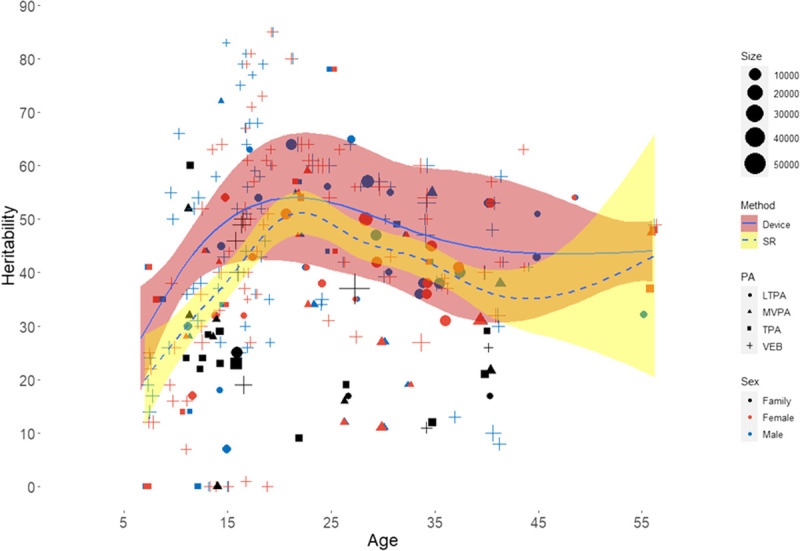
Heritability estimates for physical activity (PA) phenotypes as a function of age. Note: The scatterplot depicts 295 heritability estimates (on a 0%-to-100% scale) from 61 twin or family studies. Color coding indicates whether the estimate derives from family studies (black, note that no sex differences were tested), studies in female twins (dark red), or studies in male twins (blue). Different shapes indicate the subclass of PA measured (TPA, square; MVPA, triangle; LTPA, circle; VEB, plus). The size of the circle is weighted by square root of the sample size. The two fit lines reflect the generalized additive model (GAM) in which PA linearly depends on a set of unknown smooth functions using age as predictor. GAM estimates these nonparametric smoothers via the backfitting algorithm (50). Separate lines are depicted for device-based estimates (solid line) and self-report estimates (dashed line), which take the study weight into account. Red (device) and yellow (self-report) areas are the 95% CIs.

Even so, the heritability of PA is not “fixed,” and there is a large heterogeneity in estimates within and across studies. The heterogeneity in heritability estimates for PA phenotypes is often stipulated in reviews by statements like “estimates of heritability vary widely, from X% to Y%,” where X and Y take on intimidating large ranges like “9% to 92%” (46), or “27% to 84%” (45), or 0% to 85% (49). Although formally correct, these wide ranges misleadingly suggest that twin and family studies yield heritability estimates that have little heuristic value. As shown in Figure 5, the large heterogeneity in heritability estimates is attenuated by grouping by study design (twin vs family), sex, and PA phenotype, and an even stronger reduction in heterogeneity occurs when the age of assessment is considered. It is not surprising therefore that using an intergenerational (parent-offspring) design yields systematically lower heritability estimates than an intragenerational (twin) design.

Figure 5 also shows that slightly higher male than female estimates are seen in childhood, but after that, the sex differences are not large from adolescence onward. Device-based estimation yields higher heritability than self-report, as before. At any specific age, CIs are relatively small for studies using self-reported PA as these are based on sample sizes that are typically tenfold higher than studies using device-based PA.

Regarding age, a pattern of increasing heritability is seen from childhood to a peak in late adolescence followed by a gradual decrease in adulthood until age 40. The apparent increase in estimates after age 40 likely reflects data becoming sparser after the middle age, also indicated by widening CIs. These age trends are repeated in twin cohorts from many different countries and surprisingly robust across different types of PA (*e.g.*, total daily or confined to leisure time) and assessment by self-report or devices. The change in heritability across the lifespan can be caused by age-related changes in the shared and unique environment, in part caused by people gravitating toward environments that suit their genetic propensity. The age-related change in heritability can also reflect an increasing suppression of the genetic propensity for PA by physiological aging and related disabilities. Finally, the same genetic variants may contribute differently to PA at different ages, or different genetic variants may be expressed at different ages. This change in genetic architecture may be partly related to the substantial changes in the amount but also in the intensity and type of PA that occurs across the lifespan (51–53). For VEB, for example, team-based competitive activities strongly increase from childhood to adolescence to gradually give way to solitary recreational activities in the course of adulthood as the main source of regular exercise (53). In other words, a *true* change in the genetic effects may occur across the lifespan if different types of exercise are favored by different gene sets. The empirical testing of such hypothesis would be greatly served by access to the actual genetic variants underlying the heritability of PA traits.

## GENETIC VARIANTS FOR PHYSICAL ACTIVITY FROM GENETIC ASSOCIATION STUDIES

### Candidate Gene Studies

The early gene finding studies on TPA, MVPA, LTPA, and VEB used a candidate gene approach, based on known biology. A clear example is presented by variants involved in dopaminergic neurotransmission like the genes for dopamine receptors *DRD1*, *DRD2*, *DRD3*, and *DRD4* or for genes involved in dopamine turnover (*DBH*, *COMT*, *MAOA*, and *TPH2)*. These variants had high appeal because they have functional effects on the efficacy of neurotransmission in the mesolimbic reward system, and the corresponding genes were nominated by research on spontaneous wheel running in rodents (54,55). However, the obvious candidates in the synaptic turnover of dopamine or its receptors show equivocal association with PA phenotypes in humans, with many failures of replication (56,57). These results do not discredit a role for the neurobiology of dopaminergic reward seeking, as only few studies have specifically tested for an association of candidate genes with the reward value of PA compared with alternative activities (58). In addition, gene-gene interactions are not often tested, and genetic variants in dopaminergic transmission may interact with each other and with those in, for example, the endocannabinoid and glutaminergic systems (55,59). Nonetheless, the current state of equivocal associations with candidate genes does illustrate that selecting genetic variants focused purely on known neurotransmitter biology has not been fruitful.

In retrospect, the candidate gene strategy overestimated both the effect sizes of single genetic variants and our ability to pinpoint these variants *a priori* based on their location in the exomes or promotor regions of plausible candidate genes. Rather than capitalizing on candidate genetic variants based on biological plausibility, genetic epidemiology has embraced genome-wide association studies (GWAS) as the correct way forward (60). In a GWAS, millions of single nucleotide polymorphisms (SNPs) spread across the human genome are tested for their effect on a given trait sequentially. GWAS showed that the effect contributed by any single variant to a phenotype is tiny rather than just small, with only an increase of ~0.05 standard deviation per effect allele at best (61). Moreover, only a part of the variants that affect gene function do so by a nonsynonymous change in the amino acid coding. Instead, most functional genetic variants affect gene transcription and are often not located in, or even near, genes (62). The regulation of the expression of genes can be based on variants that are millions of base pairs away or even on remote chromosomes.

By testing a wide range of SNPs known to vary between humans, GWAS studies are free from selection of genetic variants based on existing biological knowledge and often yield results related to genes that would not have been selected based on that prior knowledge. GWAS-derived genetic variants must still demonstrate biological plausibility, but this is now done *a posteriori*, once the contribution of the genetic variant to the heritability of PA has first been firmly established.

### Genome-Wide Association Studies

Table 3 summarizes the current evidence from the eight published GWAS on PA (63–70).

**TABLE 3:** Genome-wide Association Studies (GWAS) on Physical Activity (PA) phenotypes.

Study	PA Phenotype	Genome Wide Significant SNPs	Locus	Prioritized Genes	Pathway/Tissue Enrichment
Self-report					
De Moor *et al.*, 2009 *N* = 2622 1,636,636 < *N* SNPs < 2051750 Reference NCBI, build 35	VEB	None	2q33.1; 10q23.2; 18p11.32	*DNATP6*^a^; *PAPSS2*^a^; *RP11–476 K15.1/C18orf*^a^	Not performed.
Kim *et al.*, 2014 *N* = 8842 *N* SNPs = 344,893 Reference NCBI, build 36	TPA	None	6p22.3; 7q21.3; 6p21.33; 10q21.2; 9q33.1; 10q26.2; 4q21.1; 11p15.1; 14q31.1	*CDKAL1*^a^; *TFPI2*^a^; *CCHCR1*^a^; *RHOBTB1*^a^; *ASTN2*^a^; *ADAM12*^a^; *CCNI*^a^; *PTPN5*^a^; *NRXN3*^a^	Maturity onset diabetes of the young (MODY).
Lin *et al.*, 2018 *N* = 11,093 AA + 10,684 EA 8,258,952 < *N* SNPs < 13,892,960 Reference Hg19, build 37	LTPA	None	1p36.23; 5q31.1; 14q24.1; 14q24.1	*ENO1-AS1*^a^; *SLC22A4*^a^; *APT6V1D*^a^; *MPP5*^a^	The homeostatic drive coupled with the reward system; the (development of the) capacity to perform LTPA.
Hara *et al.*, 2018 *N* participants = 16,016 7,094,228 < *N* SNPs < 11,070,774 Reference Hg19, build 37	VEB	rs10252228 (EA = G)	7p14.3	*DPY19L1/NPSR1*	No enrichment survived multiple testing.
	VEB		2q24.1	*KCNJ3–NR4A2* ^a^	
Klimentidis *et al.*, 2018 *N* participants = 377,234 *N* SNPs = 11.8 M Reference Hg19, build 37	MVPA	rs7804463 (EA = T); rs429358 (EA = T); rs2854277 (EA = C); rs7791992 (EA = C); rs3094622 (EA = A); rs149943 (EA = G); rs2035562 (EA = A); rs2988004 (EA = T); rs1043595 (EA = G)	7q33; 19q13.32; 6p21.32; 7p12.1; 6p21.33; 6p22.1; 3p12.1; 9p13.2; 7q32.1	*EXOC4;* *APOE;* *HLA-DQB1;* *C7orf72/SPATA48*^b^; *RPP21;* *ZNF165;* *CADM2;* *PAX5;* *CALU*	Adrenal gland; thyroid gland; pituitary gland; skeletal muscle; adipose tissue; brain (cerebellum, frontal cortex, anterior cingulate, amygdala, hypothalamus, nucleus accumbens, caudate, putamen, hippocampus)
Klimentidis *et al.*, 2018	VPA	rs1248860 (EA = G); rs2764261 (EA = A); rs13243553 (EA = G); rs3781411 (EA = C); rs328902 (EA = C)	3p12.1; 6q21; 7q33; 10q26.13; 7p14.2	*CADM2;* *FOXO3;* *EXOC4;* *CTBP2;* *DPY19L1*	
Klimentidis *et al.*, 2018	VEB	rs62253088 (EA = T); rs166840 (EA = G); rs10946808 (EA = A); rs159544 (EA = A); rs75930676 (EA = T); rs111901094 (EA = G)	3p12.1; 17p11.2; 6p22.2; 5q12.1; 14q24.2; 19p13.11	*CADM2;* *AKAP10;* *HIST1H1D;* *CTC-436P18.1;* *SIPA1L1;* *GATAD2A*	
Wang *et al.*, 2022 *N* participants = 606,820 19.1 M < *N* SNPs < 22.5 M (significance adjusted 10-fold to 0.5 * 10^−9^) Reference Hg19, build 35	MVPA	rs1691471 (EA = T); rs1625595 (EA = C); rs13201721 (EA = T); rs385301 (EA = C); rs1160545 (EA = T); rs142601240 (EA = AT); rs7613360 (EA = C); rs9903845 (EA = C); rs182484063 (EA = C); rs7946119 (EA = C); rs10673865 (EA = T); rs71604175 (EA = A); rs4352559 (EA = T); rs72834698 (EA = A); rs11762545 (EA = T); rs11989077 (EA = A); rs568546 (EA = T); rs1788761 (EA = A); rs6063831 (EA = A)	3p12.1; 11q13.2; 6q24.1; 17p11.2; 2q11.2; 2p25.1; 3p21.31; 17q22; 4p15.1; 11p15.2; 4q25; 5p15.31; 5q12.1; 6p22.2; 7p22.3; 8q22.3; 11q22.3; 18q11.2; 20q13.2	*CADM2;* *CD248; ACTN3;* *RN7SKP106;* *AKAP10;* *LINC01104;* *PDIA6;* *ACTBP13;* *CA10;* *MESTP3;* *SOX6;* *LEF1-AS1;* *ADCY2*^c^; *LINC02057*^c^; *H2BC5*^c^; *MAD1L1*^c^; *RIMS2*^c^; *CWF19L2*^c^; *NPC1*^c^; *LOC105372666*^c^	Brain (visual information processing and the reward system, including enrichment for dopaminergic neurons), cell signaling, wound healing, locomotion, and skeletal muscle.
Wang *et al.*, 2022	MVPA		3p12.1; 17p11.2; 3p21.31; 11p15.2; 17q22; 6q27; 2p25.1; 19p13.12; 19q13.31; 7q33; 11q13.12; 17p11.2; 3p21.31; 2q11.2; 2q11.2	*CADM2*^b^; *AKAP10*^b^; *CAMKV*^b^; *SOX6*^b^; *CA10*^b^; *PDE10A*^b^; *PDIA6*^b^; *ILF3*^b^; *NECTIN2*^b^; *EXOC4*^b^; *PACS1*^b^; *SPECC1*^b^; *MST1R*^b^; *LONRF2*^b^; *CHST10*^b^	
Device-based					
Doherty *et al.*, 2018 *N* participants = 91,105 *N* SNPs = 9,926,106 Reference Hg19, build 37	TPA	rs564819152 (EA = A); rs2696625 (EA = A); rs59499656 (EA = A)	10p12.31; 17q21.31; 18q12.3	*SKIDA1;* *KANSL1-AS1;* *SYT4/RIT2*	Adrenal gland; pancreatic; skeletal muscle; brain (amygdala, anterior cingulate cortex, basal ganglia, accumbens, caudate, putamen, cerebellum, frontal cortex, hippocampus, hypothalamus).
Doherty *et al.*, 2018	MVPA		12p13.32	*KCNA6* ^b^	
Klimentidis *et al.*, 2018 *N* participants = 91,084 *N* SNPs = 11.8 M Reference Hg19, build 37	TPA	rs55657917 (EA = T); rs59499656 (EA = A)	17q21.31 18q12.3	*CRHR1;* *SYT4/RIT2*	Adrenal gland; thyroid gland; pituitary gland; skeletal muscle; adipose tissue; brain (cerebellum, frontal cortex, anterior cingulate, nucleus accumbens).
Klimentidis *et al.*, 2018	VPA	rs743580 (EA = A)	15q24.1	*PML*	
Klimentidis *et al.*, 2018	VPA (+ VEB and VPA self-report)		3p12.1; 15q24.1; 19q12	*CADM2*^b^; *PML*^b^; *CCNE1*^b^	
Klimentidis *et al.*, 2018	TPA (+ MVPA self-report)		5q14.3; 14q32.31; 15q24.1 17q21.31	*MEF2C*^b^; *RCOR1*^b^; *STOML1*^b^; *CHRH1*^b^	
Qi *et al.*, 2022 *N* participants = 88,411 *N* SNPs = 8,951,705 Reference Hg19, build 37	TPA 6 p.m.–8 p.m.	rs301799 (EA = T); rs2909950 (EA = G)	1p36.23 5q33.1	*SLC45A1;* *NMUR2;*	Brain, blood, and immune-related mechanisms; digestive (esophagus, colon) and endocrine tissues (thyroid, testis, adrenal gland).
Qi *et al.*, 2022	TPA 8 p.m.–10 p.m.	rs2006810 (EA = T)	7q11.22	*GALNT17*	
Qi *et al.*, 2022	TPA	rs1268539 (EA = A)	9q33.3	*GAPVD1*	
Qi *et al.*, 2022	TPA 8 a.m.–10 a.m.	rs564819152 (EA = G)	10p12.31	*SKIDA1*	
Qi *et al.*, 2022	TPA 6 a.m.–8 a.m.	rs2138543 (EA = A)	12q12	*CPNE8*	
Qi *et al.*, 2022	TPA and MVPA	rs2532402 (EA = G)	17q21.31	*KANSL1*	
Qi *et al.*, 2022	TPA	rs3837946 (EA = T)	19p13.2	*PIN1*	
Qi *et al.*, 2022	MVPA		1p35.1; 17q21.31	*RN7SKP16*^b^; *KANSL1*^b^	
Qi *et al.*, 2022	TPA		1p36.23; 16q22.1; 5q33.1; 5q33.3; 10p12.31; 17q21.31; 19p13.2; 22q13.2	*RERE*^b^; *PDXPC2P*^b^; *NMUR2*^b^; *PBX3*^b^; *DNAJC1*^b^; *FMNL1*^b^*; KANSL1*^b^; *ZNF846*^b^*; PIN1*^b^; *LINC00634*^b^	

^a^ Some studies prioritized PA genes based on a more lenient threshold (10^−5^ or 10^−6^) and one or more functional annotation strategies.

^b^ Some studies prioritized PA genes based on one or more functional annotation strategies.

^c^ Genome-wide significance based on directionally consistent bivariate associations across MVPA and a sedentary behavior phenotype.

EA, effect allele increasing PA.

The first genome-wide association (GWA) study on PA that we conducted in 2009 (67) tested for an association with leisure time exercise behavior in two independent samples comprising 1644 Dutch and 978 American subjects. In retrospect, unsurprisingly, neither sample yielded results that withstood the scrutiny of the multiple testing correction that needs to be applied because of the millions of tests performed simultaneously (*P* value less than 5 × 10^−8^). Additional studies in Korea, the United States, and Japan also largely failed to detect significant associations after the required stringent correction for the multiple testing burden (63,69). Success came when GWAS was scaled up to hundreds of thousands of participants by using the unique resource of the UK Biobank (UKB) assessing various PA phenotypes with touchscreen-based surveys, among which are MVPA and VEB (66).

After applying corrections for work-related PA and an indicator of socioeconomic status, Klimentidis *et al.* (66) found associations with weekly energy expenditure in MVPA at nine loci (see Table 3 for the lead SNPs indexing these loci). A dichotomy of zero versus 3 d of 25+ min of vigorous PA yielded six loci that were associated with variation in this VPA measure. For VEB, a dichotomy of participant spending no versus at least 2 d of sports or other exercises for 15+ min added another five genome-wide significant loci, with the most strongly associated variant in the *CADM2* gene showing up in MVPA as well. In a large trans-ancestry GWAS meta-analysis on sedentary behaviors and MVPA, combining results in up to 703,901 participants from 51 studies, Wang *et al.* (70) further increased the yield for MVPA, even with a relatively “poor” PA phenotype. For a dichotomy of not engaging versus regularly engaging in 20 min·wk^−1^ of MVPA, lead SNPs from 11 loci were genome-wide significant (six not reported before), and four of these had significant reverse effects on sedentary behaviors. Bivariate association using sitting time as an additional phenotype increased the MVPA loci to 19.

The above demonstrates a main truism in the GWAS field, namely, that a scale of hundreds of thousands of participants is indeed needed to identify genetic variants in highly polygenetic phenotypes. A second truism is nicely illustrated by analysis in the UKB: increased reliability and heritability of the PA phenotype can somewhat alleviate this need for large sample sizes. Most genome-wide significant loci for PA traits so far have been found using accelerometer-derived PA measures in UKB participants, even if the subset of UKB participants that has such data is only a quarter of the full set of participants with survey-based PA measures. To date, there have been three GWA studies based on accelerometry-derived activity phenotypes in UKB (65,66,68). To be consistent with the earlier family and twin studies, the focus here is exclusively on the TPA and MVPA traits, but note that these studies also extensively looked at the genetic association with sedentary time, light PA, and sleep duration, finding significant results for these phenotypes as well (65,68).

Klimentidis *et al.* (66) extracted two measures from up to 7 d of accelerometer wear. Overall acceleration was used as a measure of TPA, and the fraction of accelerations greater than 425 mg as a measure of VPA. GWAS yielded two significant associations for TPA and one for MVPA. Doherty *et al.* (68) used a machine learning approach to extract PA phenotypes, including overall activity, sleep duration, sedentary time, walking, and moderate-intensity activity. This study identified a locus specifically associated with MVPA and two loci with TPA, of which rs59499656 near the *SYT4* gene overlapped with the locus also found by Klimentidis *et al.* (66). The third study more fully captured the complexity of 24-h PA patterns (65). It defined 27 accelerometry-derived PA measurements of which many related to circadian rhythms and sleep, active to sedentary transition probabilities, or were hard to interpret as a specific PA. Others could be more readily classified as reflecting daytime TPA and MVPA measures. GWAS in 88,411 individuals with these PA phenotypes yielded six associations with TPA, one of which (rs2532402 near the *KANSL1* gene) also influenced MVPA.

### Prioritized Genes

It is rare that the genetic variants identified by GWAS can be readily translated into a well-defined biological mechanism. The functional consequence of the effect allele in the lead SNP of an associated genomic locus is often unknown before detailed experimental follow-up has been done (71,72). To deal with this, a plethora of “functional annotation” methods is available that try to find patterns in the GWAS results, sometimes focusing on the genome-wide significant SNPs only but often casting a wider net of suggestive SNPs (*P* < 10^−6^). Many of these methods focus on prioritizing the most likely genes responsible for the association to the phenotype. These methods use gene-based association tests, identify effects of the significant SNPs on gene expression in phenotype-relevant tissues and cell types, or test for enrichment of the associated SNPs for chromatin-based annotations like promotor sites or DNase I hypersensitivity sites or contact with enhancers (73–79). A caveat of this *in silico* gene prioritization is that different methods often nominate different genes and that there is no gold standard. Therefore, triangulation is often used across a variety of gene prioritization approaches.

Table 3 gives a selection of the main genes prioritized by the GWAS studies on PA, with the clear disclaimer that a much richer set can be extracted from the (supplements to the) the original reports (63,65,66,68,70). A list of genes that appear in more than one study stand out for further scrutiny in future replication studies, which include *CADM2*, *KANSL1*, *SYT4*, and *AKAP10*. Furthermore, a number of genomic regions seem to be enriched for loci with significant association to PA in multiple studies (3p12.1; 17q21.31; 18q12.3; 17p11.2; 10p12.31; 15q24.1; 1p36.23).

The prioritized genes near significant (and suggestive) loci have been used in follow-up analysis to detect their enrichment in specific biological pathways. For PA, several pathways have been nominated, with a few standing out for their recurrence. The most often mentioned biological pathway leading to variation in PA involves the brain, with a clear emphasis on limbic structures and more specifically structures associated with dopaminergic processing of reward signals in structures like the nucleus accumbens. A second pathway relates to skeletal muscle biology, a third to the endocrine systems with the adrenal gland most mentioned, and a fourth to blood cell physiology and immune-related mechanisms. Experimental work, for example, in animal models, on these pathways and their nominating genes is required to confirm or refute their true role in PA.

### SNP-Based Heritability

Apart from yielding biological clues, GWAS summary statistics afford a set of alternative methods to estimate the heritability of a phenotype without resorting to known degrees of relatedness based on pedigree/family structure or twin zygosity. One method Genome-wide Complex Trait Analysis (GCTA) computes the genetic relatedness matrix across all SNPs for all possible pairs of participants and regresses this relatedness on the phenotypic resemblance of the pair (80). A second method uses the summary statistic from a GWAS meta-analysis to tests the regression of the linkage disequilibrium (LD) score of each SNP (reflecting how correlated it is with nearby SNPs) on the effect size of its association to the phenotype, where the slope of this regression corresponds to the SNP heritability (81). These SNP-based heritability (h^2^_SNP_) estimates will typically only be about one third of twin-based heritability estimates, because tagging SNPs on commonly used assays capture only part of the genomic variation, causing some genetic effects (*e.g.*, nontagged alleles or repeat variants, copy number variants, rare alleles with frequency <0.01, gene-sample population interactions, and nonadditive effects) to be missing in h^2^_SNP_ compared with twin-based heritability estimates (82).

Five studies computed h^2^_SNP_ for PA traits, three of which used self-reported PA (66,70) and three used device-based PA (65,66,68). SNP-based heritability proved to be systematically higher in the studies using accelerometers than in the self-report studies. Based on self-report, h^2^_SNP_ for VEB was between 3.3% and 5.6%, and for MVPA between 4.6% and 8.6%. Based on accelerometers, h^2^_SNP_ for MVPA was between 10% and 18% and for TPA h^2^_SNP_ was 21%. This higher SNP-based heritability for device-based than self-reported PA repeats the patterns seen in family and twin studies earlier and may reflect a lower measurement error in device-based measures. It could also point to different genetic variants influencing self-report and device-based measures, but this is not likely. The one study that used both self-report and device-based PA showed substantial overlap between PA loci deriving from self-report and accelerometers (66).

### Polygenic Scores

Currently, the number of genetic variants for PA that meets genome-wide significance is still modest. At first sight, this does not bode well for our ability to predict future PA behavior by measuring genetic variation. However, as was done in the computation of h^2^_SNP_, the information across all associated SNPs, even when they do not reach genome-wide significance levels, can be used to obtain meaningful genetic predictors of PA. The most used predictor is the polygenic score (PGS), also referred to as a polygenic risk score (PRS) when used in the context of disease phenotypes (83). A PGS for an individual can be computed by summing the product of the size of effect of a single effect allele (often expressed as the regression coefficient) times the amount of effect alleles that individual carries (0, 1, or 2), across all relevant genetic variants detected by the GWAS. A PGS for a PA phenotype thus estimates the predicted change in the PA phenotype compared with the population average based on all genetic variants influencing that PA phenotype.

Because international GWAS consortia adhere to Open Science principles, the relevant summary statistics of the SNP associations to PA traits (dose of effect alleles and their effect size) are almost always made freely available. This means that in any other cohort or intervention study where participants have supplied DNA, one can compute the genetic propensity for PA in these participants based on public downloadable GWAS results. This was done, for example, in two large Finnish cohorts. Participants' PGS that were based on the UKB summary statistics for both accelerometer-based and self-report MVPA successfully predicted MVPA in the independent cohorts, although the explained variance was low (84). For both self-reported and objectively measured MVPA, individuals in the highest PGS deciles of the Finnish cohorts had significantly (11%–28%) higher MVPA volumes compared with the lowest PGS deciles.

## BIOLOGICAL PATHWAYS UNDERLYING DIFFERENCES IN REGULAR PHYSICAL ACTIVITY

The robust and repeated demonstration of contribution of heritable factors to all PA phenotypes requires that models of the determinants of PA, which are now focused on the behavioral, social, and environmental pathways, incorporate the biological pathways underlying this heritability. Combining the bottom-up gene finding results above with the theory-driven nominations by the consensus paper in Medicine & Science in Sports & Exercise by the GenBioPAC consortium (47), two biological pathways that could lead from genetic variation to individual differences in PA behaviors stand out: cardiorespiratory and musculoskeletal exercise ability traits, and motivational mechanisms in the brain.

The current evidence from the field of genetics in support of these pathways comes mostly from detecting a significant overlap between the genetic factors that influence key phenotypes in the biological pathways (“intermediate” phenotypes) and the PA phenotype of interest. Presence of a significant genetic correlation between PA and intermediate phenotypes like aerobic fitness or the acute psychological response to exercise is compatible with the idea that they are part of the biological pathways leading from genetic variation to individual differences in PA. In contrast, the absence of such a significant genetic correlation — in sufficiently powered studies — directly falsifies a causal role of the biological pathway.

There are a variety of ways to detect a genetic correlation between hypothesized determinants and actual PA phenotypes. First, using multivariate extensions of twin or extended family designs, the correlation between the latent genetic factors influencing PA and the intermediate phenotype can be computed from the variance-covariance structure (14,85,86). Second, the genome-wide genotypes across millions of SNPs can be used to compute a genetic relation matrix between all individuals in the study. Using similar logic as in the twin design, using the genetic resemblance that exists even between unrelated individuals and their resemblance for intermediate and PA phenotypes can estimate the genetic correlation (87). This SNP-based method (GCTA-GREML) requires access to the individual-level genotypes in samples that assessed both intermediate and PA phenotypes. Another SNP-based method that estimates genetic covariance by using LD score regression just needs the public available GWAS summary statistics for intermediate and PA phenotypes to compute genetic correlations (88). Third, if there is genetic overlap, a polygenetic score based on genetic variants influencing the intermediate phenotype should be able to predict PA levels. For example, a genetic correlation between “liking” exercise and PA would be reflected in the polygenetic score for liking, significantly predicting actual PA levels.

### Exercise Ability

To be able to engage in regular PA, in particular in the moderate to vigorous class, requires the ability to do so. The importance of exercise ability automatically nominates genetic variants that reduce movement/exercise capability by causing congenital defects in the cardiovascular and respiratory systems (89,90), sensorimotor control systems (91), or the musculoskeletal system (92) as candidates to influence PA. However, even when overt physical disability due to rare disorders is used as an exclusion criterion, there is abundant variation in exercise ability in the general population because of more common variants. Most physical fitness traits show a textbook normal distribution across the population. It stands to reason that those who score higher on parameters like endurance, strength, flexibility, motor speed, and coordination find it easier to engage in MVPA, whereas those with lower capabilities or with (large) overweight will struggle. Given that people generally that people generally like doing what they are good at and the strong positive cultural attitudes toward being good at exercise, a reasonable expectation is that high levels of exercise ability will lead to more PA. Exercise ability should, however, not only be defined in terms of performance capacity but also in terms of being able to withstand potential injuries. A downside of being a fervent exerciser is the increased risk of sports injuries. Those with higher sensitivity to injury, possibly linked to or aggravated by being overweight, will be less motivated to engage in moderate to vigorous exercise, like sports.

Most physical fitness characteristics (strength, endurance, speed, flexibility, and balance) are known to be heritable (93,94). This heritability partly reflects innate differences in basal levels but will also incorporate genetic effects on the vast differences in the responses to a standardized training protocol. In the HERITAGE family study, Bouchard *et al.* (95) have extensively demonstrated this heritability of “trainability” for multiple exercise ability phenotypes, including V˙O_2max_, skeletal muscle enzymes, and resting and submaximal heart rate. Large differences exist in the response to exercise-induced muscle damage (96), and genetic factors have been repeatedly implicated in the susceptibility for sports injuries (97,98).

Support for a genetic overlap between exercise ability and PA phenotypes comes from bivariate modeling in twin studies that assessed physical fitness phenotypes and daily PA levels. These confirm that PA ability and PA behavior are genetically overlapping, with genetic correlations (*r*_G_) between PA and endurance capacity (V˙O_2max_) as high as 0.43 (14). Detection of this genetic correlation using SNP-based methods is currently hampered by the absence of GWAS-confirmed genes for exercise ability (99). The field seeking exercise ability genes still almost completely relies on candidate gene approaches (100–103). Despite the valid concerns about the reproducibility of these candidate genes for exercise ability, they do seem to be associated with PA. Out of the 45 candidate genes for exercise ability examined by Wang *et al.* (70), 32 carried a variant that was associated with MVPA with a *P* value of 0.01 or lower. Traditional thresholds for genome-wide significant association to MVPA were reached for three of these (*PPARD*, *APOE*, and *ACTN3*).

Interestingly, the latter *ACTN3* gene immediately demonstrates that associations between PA ability and PA behavior at the single variant level can be misleading. Extensive links to exercise ability have been shown for a common *ACTN3* variant that introduces a premature stop codon (rs1815739, R577X), but neither this variant nor nearby variants in LD with it were associated with PA phenotypes in the meta-analysis of Wang *et al.* (70). Instead, the genome-wide significant association between *ACTN3* and MVPA found was due to a previously unidentified missense variant (rs2229456) that was shown to lower maximal force production during contraction, thus providing protection from exercise-induced muscle damage. Hence, ACNT3 does not play a role in PA through its effect on exercise ability but seems to act almost entirely through its effect on injury sensitivity.

### Exercise Enjoyment

More positive affective responses to acute bouts of PA have been systematically found to predict higher levels of participation in regular PA (104,105) as does a general enjoyment of exercise and sports activities (86,106–108). That this prediction may reflect a causal effect receives support from twin studies that have unveiled a high genetic correlation between affective responding and enjoyment on the one hand, and regular engagement in PA on the other (14,109). For example, Schutte *et al.* (14,109) estimated the heritability of the affective responses during and after exercise and the overlap with the genetic factors influencing regular VEB. Genetic factors explained 15% to 37% of the individual differences in various affective responses during and after (sub)maximal exercise tests in the cycle ergometer and treadmill. Without exception, more positive affective responses were associated with higher amounts of VEB at the 2-year follow-up, and this association was accounted for by an overlap in genetic factors influencing affective responding and regular exercise behavior (0.09 < *r*_G_ < 0.40). They also observed a genetic correlation between extraversion and VEB at follow-up (*r*_G_ = 0.24). Two studies (85,106) reported significant heritability estimates for intrinsic motives for LTPA (36% to 40%) and VEB (47% in males, 49% in females). Huppertz *et al.* (85) further showed that the enjoyment of sports and exercise activities was genetically correlated (male: *r*_G_ = 0.70 ; female: *r*_G_ = 0.68) with the weekly METminutes spent on VEB. For the other side of the spectrum, “embarrassment” during VEB, substantial heritability (27% to 59%) was also shown, and embarrassment was negatively genetically correlated (−0.30 < *r*_G_ < −0.40) with the weekly METhours spent on VEB.

At the genome level, we recently tackled an individual's self-reported *liking* of PA in over 157,000 individuals from the UKB (109). GWA on self-reported liking of five PA behaviors (going to the gym, working up a sweat, exercising with others, exercising alone, and bicycling) plus an additional derived trait of overall PA liking showed significant genetic correlations with self-reported vigorous PA and strenuous VEB (*r*_G_ = 0.38–0.80) and accelerometry-derived (*r*_G_ = 0.26–0.49) PA measures in the UKB. Despite the PGS for PA liking being based on much older UKB participants, its computation in an independent younger sample allowed significant prediction, not just of PA liking but also cross-prediction of regular VEB. Moreover, four of the loci significantly influencing liking of PA (*APOE*, *CADM2*, *HIST1H1D*, and *SKIDA1*) were previously found to be associated with the actual level of engagement in PA (Table 3).

To summarize, bottom-up empirical gene finding and top-down theoretical expectations most strongly converge on brain circuitry related to the balance of punishments and rewards accrued by engaging in PA, and on the ability to perform (intense or prolonged) PA, ideally at an above-average level compared with peers (15) and without sustaining (repeated) injuries (97,98).

## GENETIC TAILORING OF FUTURE INTERVENTION PROGRAMS

The overwhelming evidence from twin and family studies — corroborated by GWAS and SNP-based heritability — that genetics make a major contribution to individual differences in PA behaviors may lead to feelings of dismay in the interventionist. If immutable genetic factors explain 50% of the variance, is our room to intervene restricted to “just” the remaining 50% environmental variance? This idea, that our ability to intervene on a phenotype may be compromised if there is a large genetic component, is widespread but mistaken. As was shown in Figure 1, the idea confuses intervention effects on the mean with those on the variance. Core risk factors for cardiac disease like blood pressure, cholesterol, and smoking all show heritability that are comparable to or even exceed that for PA (111–113). This has not prevented us in any way to successfully intervene on these factors. Interventions are about shifting the mean of the distribution toward a more favorable value, for instance, to higher levels of daily PA for all. Only when our intention is to reduce each and all individual variation in the PA levels of a population, we would run into genetics as a fierce opponent.

The above notwithstanding, it would be inappropriate to not acknowledge that, based on their genotypes, it may be harder to engage some people in PA behaviors than others. Just as in pharmacogenetics where the prescriptions of type and dose of medicine are made dependent on the genomic make-up of the individual, balancing drug efficacy and the risk for adverse events, some individuals may require different types of interventions or be guided to different types of PA. Such a personalized approach based on genotyping is an extra tool to help increase the population levels of PA, not a replacement. Proven approaches like goal setting, social support, reinforcement through self-reward, and structured problem solving remain of unabated importance, as is alerting the public to the hazards of inactivity through repeated campaigns, obligatory physical education at school, commitment of resources to safe and affordable opportunities for exercise and active transportation (mixed land use, bike lanes, and walking trails), and the training of informed PA professionals and creation of social networks that reinforce PA behaviors.

None of these proven approaches are to be abandoned, but we need to seek ways to incorporate the new genetic knowledge in these approaches to improve their success. How to achieve this? There are broadly three current strategies to use genetic information in health care: 1) use genetic risk scores to focus our resources for intervention on those who are likely to need the intervention the most, 2) give feedback on where people fall on the genetic risk scale to increase their motivation to engage in the intervention to avert disease outcomes, and 3) tailor our interventions to better fit the person's genetic risk profile.

### Focus Interventions on at-Risk Individuals

By using the PGS for PA, we could identify vulnerable individuals who are genetically predisposed to low PA and therefore may benefit more from early detection, enhanced monitoring, and more frequent guidance. This idea of focusing our limited resources for intervention and monitoring on those who may need them the most has been advanced in the field of “personalized medicine.” Whereas early GWAS findings made only modest contributions to typical metrics of clinical utility like Number of Patients Reclassified, area-under-the-curve statistic, sensitivity and specificity, and the C-index, recent increases in the scale of GWAS consortia are rapidly changing this. At least for breast cancer, type 2 diabetes, and coronary artery disease (CAD), there are PRS available with sufficient predictive power for clinical implementation (114). For example, using data from 2.1 million individuals from the Clinical Practice Research Datalink, it was estimated that adding the PRS to the recommended current guidelines to initiate statin therapy already translates to the prevention of 7% more CAD events than using conventional risk factors alone (115).

Of course, the genetic risks for CAD and breast cancer remain “unseen” until the disease becomes manifest, whereas we do not need DNA to detect who is physically active and who is not. The clear advantage of PGS over the assessment of ongoing health behaviors is that a PGS can provide an estimate of the risk trajectory across a lifetime, rather than the prediction window of a few years covered by a single snapshot of the current PA level. This means that in terms of forecasting who is at risk of becoming low physically active as an adult, a PGS can guide focused interventions in childhood and early adolescence, when the genetic propensity is not yet as visible in behavior as in late adolescence and adulthood.

### Providing Feedback on Genetic Risk

A second application of the PGS is to raise awareness in people, or their custodians, of the heightened risk of turning into a physically inactive person. The success of that approach is predicated on the availability of methods to convey this information in a digestible manner that avoids unwarranted fatalistic fears or unwarranted optimism and effectively changes their PA habits in the desired direction. A highly cited article on this topic presents a meta-analysis of studies trying to change a variety of health behaviors by informing participants of their genetic risk for disease outcomes or their risk for obesity (116). Overall, feedback on genetic testing did not change risk behaviors, including PA, with one or two exceptions (*e.g.*, more suntan use when confronted with high melanoma risk). Even more sobering, a recent study that tested whether PA assessed by accelerometers was increased after clinical and genetic risk disclosure did not detect any changes in PA behavior (117). Interestingly, by presenting null findings for effects on health behaviors, including PA, the extant literature also debunks the often voiced concern that feedback on genetic testing might lead to unintended worsening of health behaviors, by inducing anxiety and defeatism in high genetic risk individuals or a loss of discipline in low genetic risk individuals (118).

As is now widely recognized by theories of behavioral change, just providing information that a behavior is beneficial for or detrimental to health does not suffice to change that behavior. If the information on risk or protection is not paired to a concrete action plan, it will not change health behaviors. In contrast, if risk counseling is coupled to (online) health behavior coaching, it may have a more positive impact. This was illustrated by the GeneRISK study in Finland that evaluated the attitudes of 7342 middle-aged individuals upon receiving personal genome-enhanced information on 10-year CAD risk, and prospectively assessed the impact on the participants' health behavior (119). Altogether, 42.6% of individuals at high risk self-reported to have made some health behavioral change compared with 33.5% of persons at low/average risk such that a higher baseline risk predicted a favorable change, with both clinical and genomic factors contributing independently. Similar benefits of disclosing genetic risk have been seen for cancer (120). GeneRISK also further allayed the concern that communication of genetic risk induces either defeatism (high risk) or debauchery (low risk). As many as 97% believed their CAD risk to be influenced by genetic factors. Despite that belief, 99% of participants thought that they can impact on their risk through lifestyle choices, and 89% indicated that their personal risk information motivated them to take better care of their health.

### Tailoring Interventions to Genetic Risk

When we intervene on PA, we often advertise regular exercise as something that will “make you feel good, improve your cognition, buffer your stress reactivity, reduce your weight.” This generic message completely fails to consider that such benefits will not be experienced by, for example, those who struggle above their preferred intensity level to keep up with the group/expectations, those whose hypothalamus ruthlessly corrects for the increased energy expenditure, and those who lack the outgoing personality or the athletic abilities that often determine one's “rank order” in organized sports activities at school, work, and sports clubs to enjoy such activities. In short, when we advertise the benefits of PA, we assume they apply to all, whereas abundant evidence suggests that both mental and physical health effects of regular PA show large individual differences that are at least partly genetically determined (15,121).

A better grasp of an individual's innate propensity for PA as well as a better grasp on what benefits and risks regular PA will bring to a specific individual can help tailor programs to more closely fit that individual and hence improve recruitment and retention of the individual into regular PA habits. It could be particularly beneficial to decompose the PGS for PA into PGS for specific types of PA, for example, LTPA, MVPA, LPA, or even sedentary behaviors, as differential odds to engage in these types of behaviors may require differentiated intervention. Another strategy would be to focus on the potential pathways by creating a separate PGS for risk scores for aversive psychological responding to exercise, low exercise ability, or high sensitivity to injury.

Those who are expected based on their PGS to respond with “feeling good” to moderate to vigorous exercise need a different advice than those who do not have the neurobiology to enjoy exercise at high-intensity levels. For those with low PGS for PA liking, obtaining increased adoption and adherence to regular PA might be as simple as reducing exercise intensity and presenting a different or larger selection of PA activities. For those with low expected benefits in terms of weight loss, increases in aerobic performance/muscle strength, or stress reduction, a cognitive (realistic) restructuring of expected mental and physical benefits based on genotype predictions may be useful, particularly in the initial phases of the PA intervention program. More generally, a PGS pointing to low exercise ability/trainability could be used to shape the intervention such that it reduces direct comparison and competition, for example, by advising solitary over team activities, or inclusive team-based activities over competitive ones.

A PGS could also be used to identify specific risks for injury and in turn lead to adaptation of the content and build-up of exercises in training programs (98). Application of a PGS for low bone mineral density in a screening program was seen to reduce the need for application of dual energy x-ray absorptiometry by ~40% with high (>93%) sensitivity and specificity (122). It is not hard to imagine that this PGS could also help tailor PA intervention, specifically in the most vulnerable population of postmenopausal women. A person with a high sensitivity to injury to any form of PA could lead to a focus on using the appropriate warm-up, strengthen specific muscle groups, use more cross-training, and better respect one's limits. If the PGS could predict even more specifically injury risk as a function of tissue (bone, tendon, and muscle), anatomical location (ankle, shoulder, and knee), or type of PA, this would help personalize training programs to maximize performance gain while minimizing overload-induced injury risk.

## FUTURE MISSION

At this point, the examples are mere speculations. The exact strategy to optimize intervention based on genotype first requires a furthering of our current understanding of genetic differences in the propensity to engage in PA. Given the differences in genetic factors expressed across the lifespan, the potential use of the various PGS described above will likely be age dependent. Children may experience rather different enjoyment “gains” when they adopt a physically active lifestyle (enjoyment) than they do in adolescence (being good at it) or in adulthood (social and health benefits). In essence, what the genetically tailored interventions at each age should optimally look like, and if they work at all, remains largely to be discovered.

A large gap in our knowledge is caused by the near absence of PA intervention studies explicitly looking at gene-by-intervention interaction effects. Whereas most intervention studies are too small for a meaningful candidate gene approach, a PGS could explain 1%–5% of the variance in PA intervention responses. Adjusting for the PGS could help increase the power to detect the effects of PA interventions, and the PGS further allows stratified analyses in subsets of individuals with low, moderate, to high genetic propensity to engage in PA. This can be used to explicitly test if, and which, health benefits are dependent on having a more or less favorable genotype for PA.

To enable gene-by-intervention interaction testing, the only three additions that interventionist need to make to their study protocols are the explicit informed consent for biomaterial collection, a secure and qualified biobank facility, and an extra U.S.$35 per participant for the genome-wide array with bioinformatics. These are nontrivial extra efforts, but entirely doable. Paired to the increasing resource of freely available summary statistics of GWAS consortia, they would provide an unprecedented opportunity to move the field of exercise genomics forward. Such studies could show how the heterogeneity in the effects of interventions on PA adoption and the heterogeneity in the effects of PA on the health outcomes is predicted by genotypes.

## Supplementary Material

## Footnotes

^1^
These METminutes weekly are often obtained by charting the type, frequency, and duration of all sports and exercise activities done regularly using an interview or a survey. Based on listings of the age-appropriate energy expenditure for the type of activity in Metabolic Equivalents of Task (MET), weekly number of times the activity is done, and the mean duration per time in minutes, the METminutes are computed per activity. If multiple activities are performed regularly, the sum of METminutes weekly across all these activities is used.
